# Feasibility and effectiveness of the baby friendly community initiative in rural Kenya: study protocol for a randomized controlled trial

**DOI:** 10.1186/s13063-015-0935-3

**Published:** 2015-09-28

**Authors:** Elizabeth W. Kimani-Murage, Judith Kimiywe, Mark Kabue, Frederick Wekesah, Evelyn Matiri, Nelson Muhia, Milka Wanjohi, Peterrock Muriuki, Betty Samburu, James N. Kanyuira, Sera L. Young, Paula L. Griffiths, Nyovani J. Madise, Stephen T. McGarvey

**Affiliations:** African Population and Health Research Centre (APHRC), APHRC Campus, Kirawa Road, Off PeponiRoad, P.O. Box 10787, 00100 Nairobi, Kenya; Department of Food, Nutrition and Dietetics, Kenyatta University, School of Applied Human Sciences Complex, Conference Road, Room HE7, P.O. Box 43844, 00100 Nairobi, Kenya; Jhpiego, Off Riverside Drive, 14 Riverside, Arlington Block-2nd Floor, Nairobi, Kenya; PATH, ACS Plaza, 4th floor, Lenana Road, P.O. Box 76634-00508, Nairobi, Kenya; Human Nutrition and Dietetics Unit, Ministry of Health, P.O. Box 43319-00100, Nairobi, Kenya; Action Against Hunger (ACF), 4th Floor, Suite 17, Green House, Ngong Road, P.O. Box 39900-00623, Nairobi, Kenya; Department of Population Medicine and Diagnostics, Program in International Nutrition, Cornell University, Ithaca, NY USA; Centre for Global Health and Human Development, Loughborough University, Loughborough, UK; Centre for Global Health, Population, Poverty and Policy, ESRC Centre for Population Change, Faculty of Social and Human Science, University of Southampton, Building 58, Room 2001, Southampton, SO17 1BJ UK; International Health Institute, Brown University, Providence, RI 02903 USA

**Keywords:** Breastfeeding, Infant feeding practices, Child nutrition, Cluster randomized controlled trials, Kenya, sub-Saharan Africa, Rural

## Abstract

**Background:**

Interventions promoting optimal infant and young child nutrition could prevent a fifth of under-5 deaths in countries with high mortality. Poor infant and young child feeding practices are widely documented in Kenya, with potential detrimental effects on child growth, health and survival. Effective strategies to improve these practices are needed. This study aims to pilot implementation of the Baby Friendly Community Initiative (BFCI), a global initiative aimed at promoting optimal infant and young child feeding practices, to determine its feasibility and effectiveness with regards to infant feeding practices, nutrition and health outcomes in a rural setting in Kenya.

**Methods:**

The study, employing a cluster-randomized trial design, will be conducted in rural Kenya. A total of 12 clusters, constituting community units within the government’s Community Health Strategy, will be randomized, with half allocated to the intervention and the other half to the control arm. A total of 812 pregnant women and their respective children will be recruited into the study. The mother-child pairs will be followed up until the child is 6 months old. Recruitment will last approximately 1 year from January 2015, and the study will run for 3 years, from 2014 to 2016. The intervention will involve regular counseling and support of mothers by trained community health workers and health professionals on maternal, infant and young child nutrition. Regular assessment of knowledge, attitudes and practices on maternal, infant and young child nutrition will be done, coupled with assessment of nutritional status of the mother-child pairs and morbidity for the children. Statistical methods will include analysis of covariance, multinomial logistic regression and multilevel modeling. The study is funded by the NIH and USAID through the Program for Enhanced Research (PEER) Health.

**Discussion:**

Findings from the study outlined in this protocol will inform potential feasibility and effectiveness of a community-based intervention aimed at promoting optimal breastfeeding and other infant feeding practices. The intervention, if proved feasible and effective, will inform policy and practice in Kenya and similar settings, particularly regarding implementation of the baby friendly community initiative.

**Trial registration:**

ISRCTN03467700; Date of Registration: 24 September 2014

## Background

Nutrition is critical for child survival and wellbeing. Child undernutrition is a major risk factor for ill health and mortality, contributes substantially to the burden of disease in low-income and middle-income countries (LMICs) and is associated with close to half of all child deaths [1]. This is mainly due to its influence on morbidity from the major causes of child deaths including acute respiratory illnesses, diarrhea, malaria and measles [2, 3]. Undernutrition is also a major factor for loss in disability-adjusted life years (DALYs), causing 81 million (18 %) loss in DALYs in children under 5 years [2]. Furthermore, undernutrition is associated with other adverse outcomes including compromised cognitive development, scholarly achievement and future economic productivity; and higher risk of metabolic diseases later in the life course [4].

There is a growing recognition of the importance of nutrition in the first 1000 days (during pregnancy and 2 years after birth) of life with regards to child growth, health and survival [5, 6]. Poor breastfeeding and complementary feeding practices are among the prime proximate causes of malnutrition in the first 2 years of life. Breastfeeding confers both short-term and long-term benefits to the child. It reduces morbidity and mortality among infants from infectious diseases, improves mental and motor development and protects against obesity and metabolic diseases later in the life course [5–8]. In 2002, the World Health Organization (WHO) and the United Nations Children’s Fund (UNICEF) jointly developed the global strategy for infant and young child feeding (IYCF), which aims at alleviating the burden of disease, largely associated with malnutrition, among the world’s children [9]. The WHO recommends exclusive breastfeeding in the first 6 months, to meet the infant’s nutritional requirements for optimal growth, development and health. Breastfeeding should be continued up to 2 years of age or more, while timely, nutritionally adequate, safe and appropriate complementary foods should be initiated at the age of 6 months in order to meet the developing nutritional needs of the growing infant [8]. Interventions promoting optimal breastfeeding could prevent 13 % of deaths, while those promoting optimal complementary feeding could prevent another 6 % of deaths in countries with high mortality rates [6].

In Kenya, like in other LMICs, poor maternal infant and young child nutrition (MIYCN) practices are widely documented. For example, according to the 2008/09 Kenya Demographic and Health Survey, only 32 % of children are exclusively breastfed for 6 months, improving from 13 % in 2003 [10, 11]. Additionally, only 39 % of children aged 6–23 months are fed according to the infant and young child nutrition (IYCN) guidelines [12]. Consequently, substantial levels of child malnutrition and poor child health and survival have been documented in Kenya, including high levels (35 %) of stunting among children aged under 5 years [10].

To address these poor IYCN practices, the Kenyan Government developed a strategy in 2007 to promote optimal IYCN practices nationally mirroring the WHO/UNICEF global strategy for IYCN [9, 13]. The strategy is actualized mainly through the Baby Friendly Hospital Initiative (BFHI), a global initiative that promotes breastfeeding in maternity wards [14]. However, the impact of this hospital-based initiative in LMICs like Kenya is deemed to be minimal. This is because most women, especially the poor, deliver at home, [10, 15], and MIYCN practices are greatly influenced by traditional beliefs and practices. Recognizing the need to reach women at the community level, the Division of Nutrition and Dietetics in the Ministry of Health is, therefore, considering implementing the Baby Friendly Community Initiative (BFCI), a global initiative which employs the principles of BFHI at the community level. Hard evidence on the effectiveness of BFCI and how it works best in the Kenyan context are needed to create the political buy-in, budgetary allocation and effective implementation at the national level. Though the BFCI is being implemented in both LMIC and high-income countries, little evidence from evaluation of the programs exits on its effectiveness in improving MIYCN [16]. The primary goal of the proposed intervention is to change breastfeeding practices, particularly to improve the rate of exclusive breastfeeding, which is currently low in Kenya [10], despite its documented importance in child survival [1, 5]. Specifically, the study aims to pilot implementation of BFCI to determine its feasibility and effectiveness with regards to breastfeeding and other infant feeding practices, nutrition and health outcomes in a rural setting in Kenya. Specifically, we aim to determine the effectiveness of the BFCI on: (i) the proportion of infants exclusively breastfed for the first 6 months; (ii) the rate of initiation of breastfeeding within the first hour of birth; (iii) other breastfeeding practices and maternal, infant and young child nutrition (MIYCN) knowledge, attitudes and practices; and (iv) on the nutritional and health status of children aged 6 months and below. The pilot study will also investigate the experiences, and facilitating and limiting factors, associated with the implementation of the BCFI.

The primary hypothesis is that implementation of the BFCI will improve access to counseling and support on maternal, infant and young child nutrition to mothers, and lead to higher knowledge and self-efficacy in breastfeeding practices, thereby resulting in adherence to WHO guidelines for breastfeeding. This is expected to lead to improved rates of exclusive breastfeeding for 6 months and other optimal breastfeeding practices. Eventually, improved practices are expected to impact on child nutritional and health outcomes in the community. While complementary feeding is very important, this study will not explore effectiveness of the intervention on complementary feeding. This is because due to financial limitations, we will only be able to follow children until 6 months of age, when complementary feeding is expected to be introduced.

## Methods

### Study setting

The study will be conducted in Koibatek sub-County, one of the six sub-counties in Baringo County of the North Rift region of Kenya. Residents in Koibatek practise mixed farming in an area that covers 2,306 km^2^. The total population for 2014 (as reported by sub-county Health Registry and Information Office) is 125,637 with 30,203 being women of childbearing age (15–49) and 4,799 of them being children under 1 year. Koibatek sub-county is subdivided into four administrative divisions (Eldama Ravine, Timboroa, Esageri and Torongo). The sub-county is mainly inhabited by the Tugen people, a Nilotic group whose main occupation and economic activity is mixed farming. The sub-county is served by 32 health facilities (26 dispensaries, 5 Health Centers and only 1 sub-county/district hospital) most (close to 90 %) of which are run by the government. The sub-county lies within the Rift Valley region where, according to the 2008/09 Kenya Demographic and Health Survey, the median duration of exclusive breastfeeding, was 1.7 months, which was among the highest in the country, being higher than the national rural average of 1.0 month. Slightly over a third of children are stunted, 20 % are underweight, while only a third of women deliver at a health facility [10]. Other evidence indicates that the prevalence of exclusive breastfeeding for 6 months is 32 % [17].

### Study design and randomization

The study will combine both qualitative and quantitative methods to achieve the study objectives. A formative study using participatory action research design will first be conducted. Then, a cluster randomized trial utilizing both qualitative and quantitative data collection methods will be conducted. The purpose of the formative qualitative study will be mainly to understand the local contexts and cultural factors that influence maternal nutrition, breastfeeding and other infant and young child feeding practices. This information will inform the adaptation of the intervention to the local contexts, and aid understanding of the potential barriers and facilitating factors to the implementation of the intervention. Another purpose of the formative study is to inform the finalization of quantitative data collection tools. Qualitative methods will be used at the formative stage as well as during and after the intervention to determine experiences with the intervention. Quantitative methods will mainly serve to determine effectiveness of the intervention with regards to breastfeeding practices and nutritional status.

**Fig. 1:**
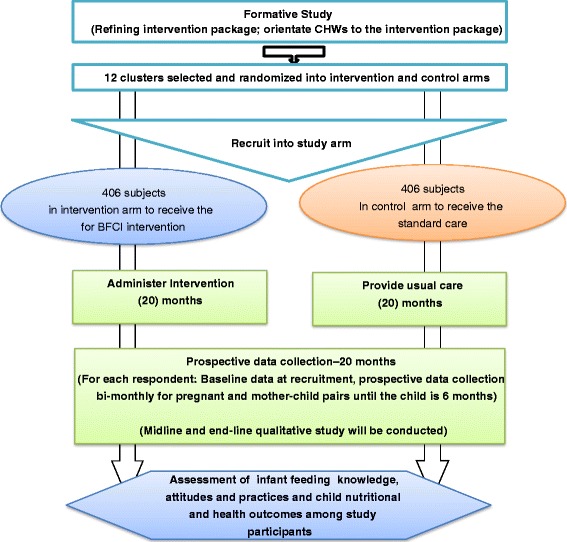
Schematic of the study design

The study will adopt a cluster-randomized trial design [18, 19]. For pragmatic purposes, community units (CUs), as defined by the government’s Community Health Strategy [20] will be used as the clusters. Thirteen CUs have been defined in the study areas. Twelve of the CUs will be randomized with half allocated to the intervention and the other half to the control arm without matching. The random sequence of allocation of the CUs to the intervention or control arm will be computer-generated. Cluster randomization is preferred over individual-level randomization to minimize contamination and for pragmatic purposes in case of future scale-up of the intervention. Randomization will be done by a data analyst who is not a primary member of the study team. Fig. 1 shows the study design schematically.

### Clusters and study populations

Twelve CUs will constitute the clusters to be included in the study. CUs are geographically defined units, mostly equal to a village (in our study setting) and usually have a population size of approximately 5000 people. The CUs are defined by the Community health Strategy, a government community-based approach where community health workers (CHWs), currently referred to as community health volunteers (CHVs) in Kenya, provide health care services to people at the community level. The reason for choosing to work with the defined CUs is pragmatic because they are administrative areas that are defined by the health care system and any intervention moving forward in the future would need to map onto these administrative units to be effective [20].

This trial will include women of reproductive age (15–49 years) who are pregnant at the time of recruitment, and their respective children from the pregnancies aged less than 6 months in Koibatek sub-county in Baringo county. These will be recruited during pregnancy on a rolling basis until the desired sample size is achieved. The target is to recruit the women as early as possible during pregnancy, particularly during the first or second trimester, so as to get as much exposure to the intervention during pregnancy as possible.

Women of reproductive age, who will have given birth before receiving at least one personalized counseling session by our trained CHWs regarding exclusive breastfeeding will be excluded. To be dropped from the analysis will be: (i) women who lose their pregnancy and/or have a still-birth; (ii) women who cannot be traced for follow-up during pregnancy; (iii) mother-child pairs of children with disability that would make their participation in the intervention difficult: for example, blind, deaf and intellectually impaired individuals or children with cleft lip.

### Recruitment

Recruitment of the study participants will be done through identification of pregnant women by CHWs, through identification by antenatal care providers in the study area, and through use of community informants to ensure high coverage. All known pregnant women in the two study areas will be invited to participate in the study until the desired sample size is achieved. Recruitment is expected to be for a period of approximately 1 year. Before the beginning of the study, a rigorous community mobilization involving community leaders and community members will be done to inform the community of the study, and to encourage pregnant women to identify with the CHWs as early as possible in both the intervention and control arm. Recruitment will last approximately 1 year from January 2015 to December 2015.

### Sample size considerations

The sample size determination was undertaken considering the cluster randomized study design [21]. An estimated sample size of 738 mother-child pairs will be required for both intervention and control arms so as to have adequate power to detect an increase in exclusive breastfeeding for 6 months from 32 % (the baseline rate of exclusive breastfeeding in the study setting) [17] to 50 %; an approximately 18-percentage point increase, although higher increases have been documented in similar interventions elsewhere in the developing world [22]. We used a level of precision of 5 % (for a 2-sided *t* test) and power of 80 %. We then adjusted for expected design effect using a design effect of 3.15 calculated based on intracluster correlation coefficient of 0.035 from another study in Kenya (unpublished) and an average cluster size of 62.5. We allowed for 10 % potential loss to follow-up. The estimated sample size is 812. We therefore expect to recruit 406 women in each study arm. Twelve CUs will be required for the estimated sample size.

### Intervention

The intervention will involve implementation of the BFCI in the intervention clusters. The proposed BFCI in Kenya is a multifaceted program for promotion of optimal breastfeeding and infant and young child nutrition, and other practices including maternal nutrition in the community. The BFCI is based on the principles of the BFHI, but extends them to the community in order to provide women with a comprehensive support system to improve breastfeeding practices and other maternal, infant and young child nutrition practices at the community level. The BFCI package (unpublished) adapted for implementation in Kenya involves an 8-step plan as illustrated in Table 1.

**Table 1:** Steps in the proposed Baby Friendly Community Initiative (BFCI) program in Kenya

Step	Description
Step 1	Have a written MIYCN policy summary statement that is routinely communicated to all health providers, community health volunteers and community
Step 2	Train all health care providers and community health volunteers in the knowledge and skills necessary to implement the MIYCN policy
Step 3	Promote optimal maternal nutrition among women and their families
Step 4	Inform all mothers and their families about the benefits of breastfeeding and risks of artificial feeding
Step 5	Support mothers to initiate breastfeeding within the first hour of birth, establish and maintain exclusive breastfeeding for first 6 months
Step 6	Encourage sustained breastfeeding beyond 6 months to 2 years or more alongside timely introduction of appropriate, adequate and safe complementary foods
Step 7	Provide a welcoming and conducive environment for breastfeeding families
Step 8	Promote collaboration between health care staff, maternal, infant and young child nutrition support groups and the local community

*MIYCN* maternal, infant and young child nutrition

CHWs (including traditional birth attendants (TBAs)) and health care professionals at the lower-level health facilities (dispensaries (level 2) and health centers (level 3)) in the participating intervention CUs will be trained on the BFCI package at the beginning of the intervention followed by on-job training and mentoring through supportive supervision by the research team and the sub-county Nutrition Officer quarterly to ensure proper implementation of the BFCI at the facility and community levels. Training materials for CHWs and health professionals will include the IYCF Counseling Package developed by UNICEF in partnership with other organizations, which has been adopted by the Ministry of Health, Kenya [23]. The package is designed to equip primary health care staff to be able to support mothers and other caregivers to optimally feed their infants and young children. The CHWs and primary care staff will be equipped with infant and young child feeding counseling cards; brightly colored illustrations that depict key infant and young child feeding concepts and behaviors to share with mothers, fathers and other caregivers. The package will be adapted to include counseling messages on maternal nutrition.

As part of the BFCI package, community support groups for mothers comprising about 20 mothers per group and including other people in the community that may support the mothers such as a CHW, a community health extension worker, an older woman and a community leader, will be formed in the intervention areas. The mothers in the group will meet regularly: for example, once a month to offer each other peer-counseling and support with regards to breastfeeding and other maternal, infant and young child nutrition practices. The CHW will be the facilitator of the group. The older woman will be a model mother, carefully selected, based on knowledge and experience with infant feeding, and will be a resource for the group. The community leader, who may be the area chief or village elder, will also be a resource person particularly to offer support on administrative issues. The extension worker, who may be a skilled nurse will offer technical advice to the group.

CHWs in both intervention and control areas will be given a motivation package given as a seed grant to the whole group of CHWs to start an income-generating activity, and training on income-generating activities.

The intervention group will also receive the BFCI package including: (i) personalized home-based counseling and support on optimal MIYCN practices by CHWs, and professional counseling of mothers by health professionals at health facilities; and (ii) formation of community support groups for mothers. In addition, they will receive MIYCN education materials. The control group will have the usual care only (Table 1). Usual care will include routine services offered to mothers and their children through the health care system including information materials regarding MIYCN, standard counseling on antenatal and postnatal care, appropriate tests during pregnancy, health facility delivery, general nutrition, hygiene, and immunization. Those in the control arm will receive routine visits by CHWs as provided for within the Community Health Strategy (usual care). Table 2 outlines the intervention package.

**Table 2:** Services and materials provided to intervention and control groups

Intervention group	Control group
a) Distribution of MIYCN educational materials (usual care)	a) Distribution of MIYCN educational materials (usual care)
b) Supportive supervision (scheduled regular visits to assess implementation of BFCI package)	b) Supportive supervision (usual planned visits by DHMT)
c) CHW motivation package (provision of a monthly stipend (seed money for an income generating activity as a group) and training for income-generation activities as incentive)	c) CHW motivation package (Seed money for an income generating activity as a group same as in the intervention group)
d) Orientation, and continuous on job training and mentoring of the HWs and CHWs/CHEWs on BFCI package implementation	
e) Formation and support of mother support groups in the community	

*BFCI* Baby Friendly Community Initiative, *CHEWs* community health extension workers, *CHWs* community health workers, *DHMT* district health management team, *HWs* health workers, *MIYCN* maternal, infant and young child nutrition

Counseling of mothers will be initiated during pregnancy as soon as the mother is recruited and will be continued until the infant is 6 months. Counseling will encompass maternal nutrition, skin-to-skin contact between mother and baby immediately after birth, immediate initiation of breastfeeding after birth, breast positioning and attachment, exclusive breastfeeding, frequency and duration of breastfeeding, expressing breast milk, storage and handling of expressed milk and lactation management. It will also focus on age-appropriate complementary feeding, starting at 6 months: age appropriate complementary foods (nutritious, safe, affordable, and locally available), feeding frequency and quantity, and appropriate feeding practices including hygiene and responsive feeding behaviors, which encourage mother-child interaction during feeding [24, 25]. However, effectiveness of the intervention on complementary feeding will not be evaluated in this study. For the intervention arm, CHWs will visit the pregnant woman about once every month up to week 34, after which they will visit the mother weekly until delivery. After delivery, they will visit the mother weekly in the first month, then once a month until 6 months. The CHWs to be involved will be existing CHWs within the existing CUs in the region under the Community Strategy. In line with guidelines of the Community Health Strategy in Kenya, each CHW will serve approximately 20 households. HIV-infected women will not be excluded nor their status identified in the data collection. The counselors in the intervention arm will be trained on the messages for both HIV-negative and HIV-positive mother with regards to infant feeding. The information materials given to both intervention and control arms will also stipulate information on feeding for HIV-exposed infants. The CHWs will further be told to inform the mothers that should they be HIV-positive, they should seek further counseling and support from health professionals/PMTCT program. An outline of the content of the counseling messages is given in Table 3.

**Table 3:** Content of counseling messages

• Maternal nutrition:	
	Food portions during pregnancy and lactation
	Appropriate foods (nutritious, affordable, and locally available) during pregnancy and lactation
	Frequency of feeding during pregnancy and lactation
• Breastfeeding:	
	Breast positioning and attachment
	Immediate initiation of breastfeeding after birth
	Exclusive breastfeeding for 6 months
	Frequency and duration of breastfeeding
	Expressing breast milk, storage and cup feeding
	Dealing with breast conditions
	Breastfeeding for HIV-positive women
• Complementary feeding:	
	Timely initiation of complementary foods
	Appropriate complementary foods (nutritious, affordable, and locally available)
	Feeding frequency and quantity
	Appropriate feeding practices including hygiene and responsive feeding behaviors
	Safe preparation and storage of foods

A formative qualitative study will be conducted before the roll-out of the intervention to inform the design and components of the intervention including content of the counseling messages. Interviews will be conducted with: (i) key informants in the study communities such as community leaders, CHWs, TBAs and health professionals; (ii) women who are currently pregnant, breastfeeding or mothers of children under 5 years. Additionally, consultations will be held with key organizations including the Division of Nutrition and Dietetics and the Division of Community Health Services in the Ministry of Health; UNICEF and other organizations working on MIYCN issues. The information gathered will be used to adapt the counseling messages and information materials. The formative study will also establish mechanisms for successfully engaging CHWs into the study.

Rigorous monitoring of the intervention will be done to ensure that the intervention is delivered as required. Process evaluation of the intervention will be done at mid-term and at end-line using the assessment tools specifically developed for the purpose. Exit interviews will be conducted with pregnant women and mothers of children aged less than 6 months at the health facilities in the intervention CUs on the counseling on MIYCN received. Another set of similar interviews will be conducted at the community level in the participating CUs. Additionally, interviews will be conducted with CHWs and health care professionals: for example, to establish if they actually obtained training on MIYCN. Further, observations will be done to determine whether BFCI is being implemented as planned: for example, if there is a written policy summary statement at the participating health facilities that is routinely communicated to health care workers. Process evaluation will lead to determination of whether the health facility should be certified as baby friendly or not as outlined in the BFCI assessment protocols. As part of monitoring of the intervention on a daily basis, mother’s diary (to record counseling sessions and content of counseling) and CHWs’ reporting tools will be used. The CHWs will be expected to submit weekly reports of their activities to the field coordinator. Close supervision of the activities of the CHWs will be done through regular spot checks and sit-in sessions by the field coordinator.

## Assessment

### Primary outcome

The primary outcome measure is the proportion of children being exclusively breastfed for the first 6 months.

This will involve determining the effectiveness of the BFCI intervention on the level or proportion of children being exclusively breastfed for the first 6 months. The advice and support received by mothers through the intervention is expected to lead to self-efficacy with regards to breastfeeding and effective breastfeeding, hence adherence to WHO guidelines on breastfeeding, resulting in improved levels of exclusive breastfeeding for 6 months. Data on breastfeeding practices will be collected longitudinally from birth every 2 months through an interviewer-administered questionnaire to the mother (24-hour recall at 2, 4, 6 months) with probes on the age at introduction of foods or liquids (if appropriate). Analyses will focus on the differences between the two study arms in the proportion of infants being exclusively breastfed at 6 months, as well as at the two earlier times of 2 and 4 months postpartum.

### Secondary outcomes

#### Qualitative

(i) Norms and cultural factors that influence breastfeeding and other maternal, infant and young child feeding practices. Data will be collected through qualitative interviews with mothers, fathers, community leaders, TBAs, CHWs, other community members, and health care providers
(ii) Enabling factors and barriers. Data will be collected through qualitative interviews with mothers, fathers, community leaders, TBAs, CHWs, other community members, and health care providers to identify key players and structures in the community that would facilitate implementation of BFCI; factors that influence uptake of interventions in the community; and any potential hindrances to the success of the intervention (for example, myths, beliefs)

#### Quantitative

(iii) MIYCN knowledge, attitudes and practices according to WHO recommendations on breastfeeding [9]. Data will be collected through self-reports by mothers using an interviewer-administered questionnaire at recruitment and every 2 months during the follow-up period to determine change in knowledge, attitudes and practices with the intervention
(iv) Timing of initiation of breastfeeding. Data will be collected through self-reports by mothers using an interviewer-administered questionnaire within the first month of birth
(v) Interventions aimed at optimal infant breastfeeding practices have been found to reduce malnutrition among infants and young children. [26]. It is, therefore, expected that the proposed intervention will have an effect on the levels of stunting, underweight and wasting. Anthropometric measurements: weight, length and mid-upper arm circumference (MUAC) will be collected on the child every 2 months during the follow-up period (months 2, 4 and 6). All anthropometric measurements will be carried out by the study staff according to standard procedures [27]. For determination of underweight, stunting, and wasting, weight-for-age *z*-scores (WAZ), length-for-age *z*-scores (LAZ) and weight-for-length *z*-scores (WLZ), respectively, will be generated using the WHO 2006 growth standards [28]. Stunting will be determined as LAZ < −2, underweight as WAZ < −2 and wasting as WLZ < −2 [29]
(vi) Evidence indicates that breastfeeding is preventive against infections such as rotaviral diarrhea [30]. It is, therefore, expected that promotion of exclusive breastfeeding would impact on the rate of diarrhea morbidity. Data on the presence of diarrhea morbidity in the last 2 weeks for the child will be collected longitudinally through an interviewer- administered questionnaire to the mother every 2 months during the follow-up period (months 2, 4 and 6)
(vii) Satisfaction with the intervention, facilitating and limiting factors. Data will be collected through self-administered questionnaire to the mother. Additionally, qualitative interviews will be conducted with the mothers and community members including CHWs on experiences with the intervention

### Data collection and analysis

The study will include both qualitative and quantitative standard data collection procedures. Table 4.

**Table 4:** Data collection outline

Formative study		
Study objective	Data/variables	Method
1. To establish local contexts and norms, which influence Maternal, Infant and Young Child Nutrition (MIYCN) practices in order to tailor the intervention package to the local communities	Attitudes and practices regarding breastfeeding and other MIYCN practices; cultural and social factors and norms that influence MIYCN	Focus group discussions (FGDs) with community members (women and men and community health workers); key informant interviews (KIIs) with key informants including community administrators and other leaders (e.g. religious group leaders), traditional birth attendants (TBAs); KIIs with facility health workers, CHWs and Sub-County Health Management (ScHMT) team
2. To identify enabling factors and barriers that may influence the implementation of BFCI and potential ways of addressing them	Key players and structures in the community that would facilitate implementation of BFCI (key influential people in the community, community resource persons that would be involved in the implementation of the project, other useful community resources); factors (e.g. socioeconomic, maternal age, social/family support) that influence uptake of interventions in the community; any potential hindrances to the success of the intervention (e.g. myths, beliefs)	Key informant interviews (KIIs) with key informants including community administrators and other leaders (e.g. religious group leaders), traditional birth attendants (TBAs); KIIs with facility health workers, CHWs and (ScHMT) team, FGDs with mothers and fathers
Cluster randomized trial		
Study objective	Variable/Data	Methods
Primary objective		
1. To determine the effectiveness of the BFCI on the rates of exclusive breastfeeding for the first 6 months	Duration of exclusive breastfeeding as a *derived variable*: age of child, current status of breastfeeding (exclusive, mixed feeding, not breastfeeding) with probes on the age at introduction of other foods or liquids (if appropriate), and a 24-hour recall feeding question	Quantitative: questionnaires to mothers and 24-hour recall at 2, 4 and 6 months;
Secondary objectives		
2. To determine the effectiveness of the BFCI on breastfeeding and other MIYCN knowledge, attitudes and practices	Breastfeeding and other MIYCN knowledge, attitudes and practices including breast positioning attachment, and frequency of feeding	Quantitative: questionnaires to mothers (at baseline and end-line)
3. To determine the effectiveness of the BFCI on rate of initiation of breastfeeding within the first hour of birth	Timing of initiation of breastfeeding with specific prompts on whether infant was put to breast immediately, within 1 hour or later, reasons for the delay in initiation of breastfeeding	Quantitative data gathered from interviews with mothers within 2 months of the birth of the child
4. To determine the effectiveness of the BFCI on nutritional and health status of children aged 6 months and below.	Anthropometric measurements including MUAC, weight and length morbidity from diarrhea using 14- day recall	Quantitative: using questionnaires, MUAC tapes, electronic weighing scale and measuring board at 2, 4 and 6 months
5. To determine satisfaction with the intervention, and the enabling factors/barriers associated with the implementation of the BCFI	Facilitating factors and barriers associated with intervention; Client satisfaction, experiences with intervention and with the processes of delivering it	Quantitative data: Likert scale (satisfaction) at end-line Qualitative: interviews with mothers, community opinion leaders, HWs and CHWs (experiences, facilitating factors and barriers) at midline and end-line

*BFCI* Baby Friendly Community Initiative, *CHWs* community health workers, *HWs* health workers, *MUAC* mid-upper arm circumference

### Qualitative data collection before, during and after the intervention

The qualitative study will involve focus group discussions (FGDs), key informant interviews (KIIs) and in-depth interviews (IDIs) before the intervention, during the intervention and at the end of the intervention. During the formative qualitative study, IDIs (*n* = ~10), FGDs (*n* = ~10) and KIIs (*n* =~20) will be conducted before the beginning of the intervention to: (i) establish knowledge, attitudes and practices regarding maternal nutrition, breastfeeding and complementary feeding; (ii) establish contextual and cultural factors, which contribute towards MIYCN practices; and (iii) inform customization of BCFI including the role of key persons involved in reproductive health such as TBAs.

Focus group discussions will be conducted with women of reproductive age (15–49 years) who are either pregnant, breastfeeding or have ever breastfed and with CHWs and village elders. Key informant interviews will be conducted among health care professionals, sub-County Health Management Team members, TBAs and other community leaders including the chief, village elders and religious leaders. This information will inform the customization of the intervention and the finalization of the quantitative tools for assessing the study outcomes. Key informants will be selected based on their social standing and knowledge of the community in terms of culture and other practices. To identify such individuals, we will talk to the administrative leaders including the chief and the village elders, who will also be considered key informants. These individuals will be included in the formative study since they are in a position to provide useful insight in the structure and ways of the community.

Qualitative data will also be collected during the intervention and at the end of intervention to document experiences and satisfaction with the intervention, challenges and enabling factors, and recommendations for change or future practice. This will be collected through IDIs and FGDs with mothers; and FGDs with CHWs and village elders; and KIIs with other community leaders including religious leaders, health care professionals, TBAs and the Sub-County Health Management Team members.

The qualitative interviews will be tape-recorded after obtaining consent from the participants. Digital recorders will be used for data collection together with notes that will be taken during the interviews. The recorded data will be transcribed verbatim to enhance accuracy.

### Qualitative data analysis

Qualitative data will be transcribed verbatim and coded in NVIVO (QSR International Pty Ltd., Burlington, MA, USA), to identify primary and meta codes and major themes. Themes: for example, regarding breastfeeding and infant feeding beliefs and norms will be identified, with attention to contradiction and diversity of experiences and attitudes. Analysis across all transcripts will be done thematically [31].

### Quantitative data collection

Quantitative data for effectiveness evaluation will be collected at baseline and every 2 months during the follow-up from all mother-child pairs in both intervention and control arms. The target population will be pregnant women and their respective children aged less than 6 months. Baseline data will be collected at recruitment, then follow-up data will be collected every 2 months as appropriate until the respective child is 6 months.

As stated earlier, data collection will involve self-reports by the mother using an interviewer-administered questionnaire, and objective measures through anthropometry (weight, length, MUAC).

Other than the data for the outcomes outlined above, contextual data including household food security, water, sanitation and personal hygiene, antenatal care, delivery characteristics, vaccination, maternal socio-demographic characteristics and socio-economic status (SES) will also be collected using an interviewer-administered questionnaire, administered by a carefully trained research assistant.

Details of quantitative data collection are outlined in Table 4.

### Statistical analysis

Analyses will involve comparison of outcomes between the intervention group and the control group using multilevel linear logistic regression and multinomial logistic models that account for clustering.

Determination of differences between the 2 trial arms in the primary outcome (exclusive breastfeeding) will be done at month 2, month 4 and at month 6. Comparison for other outcomes such as morbidity from diarrhea and malnutrition will also be done at months 2, 4 and 6. Analyses will involve a comparison of differences in the intervention and control groups with regard to the primary outcome (exclusive breastfeeding) and secondary outcomes (other infant feeding practices, diarrhea morbidity and nutritional outcomes), controlling for baseline measures (for example, previous breastfeeding practices) and prognostic factors (for example, SES and household food security) if randomization of the clusters does not control for differences in these factors at baseline, through analysis of covariance (ANCOVA). Statistical methods to determine change will include multilevel linear regression for continuous variables (for example, z-scores, multinomial logistic regression analysis for categorical variables: for example, whether exclusively breastfed at 6 months, 4 months or 2 months [32]. Intention-to-treat analysis [33] will be applied as appropriate. Statistical analysis will mostly be done using Stata (StataCorp, College Station, TX, USA). (See Table 5 for detailed outline of data analysis plan).

**Table 5:** Data analysis outline

Formative study				
Study objective	Primary outcome variables	Independent variable	Control variables	Type of analysis
1. To establish local contexts and norms, which contribute to maternal infant and young child nutrition (MIYCN) practices so as to customize the intervention package	Factors that influence breastfeeding and other MIYCN practices	Not applicable	Not applicable	Thematic
2. To identify facilitating factors and barriers that may influence the implementation of BFCI	Facilitating factors and barriers to the implementation of BFCI	Not applicable	Not applicable	Thematic
Cluster randomized trial				
Study objective	Outcome variables	Independent variable	Control variables	Type of analysis
1. To determine the effectiveness of the BFCI on the rates of exclusive breastfeeding for the first 6 months	Exclusive breastfeeding (EBF) at 6 months, 4 months and 2 months	Intervention status	SES, food security, water and sanitation, maternal characteristics, antenatal care	Multinomial logistic regression
2. To determine the effectiveness of BFCI strategy on MIYCN knowledge and attitudes	a. Knowledge levels on MIYCN	Intervention status	SES, food security, water and sanitation, maternal characteristics, antenatal care	Logistic regression
	b. Attitudes on MIYCN			
3. To determine the effectiveness of the BFCI on rate of initiation of breastfeeding within the first hour of birth and its implications on successful EBF for the first 6 months	Initiation of breastfeeding within 1 hour	Intervention status	SES, food security, water and sanitation, maternal characteristics, antenatal care	Logistic regression
4. To assess the change in nutritional and health status among infants and young children from the implementation of BFCI	a. Stunting	Intervention status	SES, food security, water and sanitation maternal characteristics, antenatal care, vaccination status	Logistic regression for stunting, underweight and wasting
	b. Underweight			ANCOVA for *z*-scores and multilevel modeling for diarrhea morbidity
	c. Wasting			
	d. *z*-scores			
	e. Diarrheal and other morbidity			
5. To determine the factors and barriers associated with the implementation of BCFI and how to address them	a. Satisfaction with intervention	Not applicable	Not applicable	Descriptive (Outcome 1)
	b. Experiences with intervention			Qualitative: Thematic analysis (Outcomes 2 and 3)
	c. Limiting and enabling factors			

*ANCOVA* analysis of covariance, *BFCI* Baby Friendly Community Initiative, *SES* socio-economic status

### Ethical considerations

Ethical approval has been granted by the Kenya Medical Research Institute (KEMRI), a recognized Ethical Review Committee, approved by the Government of Kenya, reference number: KEMRI/RES/7/3/1-NON-SSC protocol number 443. The investigators will uphold the fundamental principles regarding research on human subjects: respect for persons, beneficence and justice. For all data collection activities, informed consent will be obtained from all the eligible participants following full disclosure regarding the study before data collection is done. Proxy consent for children will be obtained from their mothers.

## Discussion

This paper describes the protocol for a cluster randomized trial whose aim is to determine the feasibility and effectiveness of the BFCI with regard to breastfeeding and other infant feeding, nutrition and health outcomes in a rural setting in Kenya. Breastfeeding and optimal infant and young child feeding promotion is an important intervention for child-survival; however, it is not yet clear which strategies are the most effective. Studies have indicated the effectiveness of counseling programs within primary health care in improving breastfeeding and other infant feeding practices particularly in high-income and middle-income countries, but little such evidence exists in low-income countries [22, 34–37]. Though the BFCI is being successfully implemented in LMICs such as Cambodia and the Gambia, little evidence from evaluation of the programs on its effectiveness in improving MIYCN exists [16]. Further little evidence exists on its feasibility in Kenya. The importance of this study is to provide the needed evidence on feasibility of implementing the BFCI in Kenya and its potential effectiveness.

The BFHI, a WHO and UNICEF global program launched in 1991 to protect, promote, and support breastfeeding in maternity wards has been found effective in improving breastfeeding practices particularly in high-income and middle-income countries [38–41]. Of concern, however, is the fact that the BFHI mainly focuses on promoting breastfeeding in the hospital setting around the time of delivery, yet in some countries including Kenya, particularly in rural settings, many women deliver at home [15]. Studies have found that while interventions that involve counseling/support at the health facility level are effective, there is evidence to suggest that combining hospital-based counseling/support with home-based visits is more effective. For example, in a study done in Brazil that compared 2 systems of delivery of breastfeeding counseling/support: a purely hospital-based system and a hospital- based system coupled with home-visits, the hospital-based intervention achieved a high rate (70 %) of exclusive breastfeeding during the hospital stay, but this was not sustained after discharge from hospital; at 10 days, this rate had dropped to 30 %. The rate of exclusive breastfeeding from 10 days to 6 months was higher in the group that received home visits (45 %) compared to the group with only hospital-based intervention (13 %) [42]. This study will provide evidence on the effectiveness of coupling home-based counseling with health facility-based counseling within primary health care facilities in a rural setting in Kenya.

Support to the mother at the community level is critical as a link between initiation of breastfeeding and continued breastfeeding and other infant and young child feeding practices, and is recommended by the WHO and UNICEF [14, 43]. Different forms of support for mothers in the community have been described and explored. These include counseling by lay CHWs, peer support groups run by trained mothers, mother support groups run by women in collaboration with health/nutrition professionals, and mother-to-mother support groups run by mothers [44–46]. Different forms of support will be differently effective in different contexts, hence the need for context-specific adaptation of the type of support through formative research in the study context [45]. We will conduct formative studies to adapt the intervention to the local context for example with regards to the counselling on nutritious, locally available foods. We will use CHWs to counsel mothers at the household level. The effectiveness of CHWs in health care delivery, particularly in child survival programs has been documented [35, 47, 48]. A study involving a non-randomized design in rural Kenya to determine the effectiveness of the government’s Community Health Strategy (that involves use of CHWs to promote health in the community) found that the strategy improved the prevalence of exclusive breastfeeding from 20 % to 52 % [35]. In the proposed study, to enhance sustainability, we propose to introduce income- generating activities for CHWs through training on income generating activities and a seed grant to start off the income generating activities . As part of support for mothers, we also propose support groups for mothers in the community that incorporate various people in the community likely to support the mothers, other than the mothers themselves, including a CHW, a health professional, an older woman and a community leader. Establishment of support groups for mothers in the community is expected to enhance sustainability of the intervention beyond the project timespan.

The timing of the counseling/support has been found to be important. Prenatal and postnatal breastfeeding counseling interventions, whether alone or in combination have been found effective in improving breastfeeding practices including duration of exclusive breastfeeding and any breastfeeding [36, 37]. In a systematic review of effectiveness of breastfeeding interventions that involved 38 trials [36], prenatal breastfeeding interventions significantly increased the rate of any short-term breastfeeding (1–3 months) rate by 39 % compared to the usual care while combined prenatal and postnatal interventions significantly increased both the rates of intermediate (4–5 months) and long-term (6–8 months) any breastfeeding compared to usual care by 15 % and 33 % respectively. Postnatal interventions significantly increased the rates of short-term exclusive breastfeeding (1–3 months) by 21 %. In another systematic review that involved 20 trials, only interventions with a post-natal component were found to be effective in improving breastfeeding practices while there was no evidence to suggest effectiveness of antenatal counseling/support [37]. This study, which involves home-based and facility-based counseling/support during pregnancy and 6 months following delivery, will provide further evidence on the effectiveness of combined antenatal and postnatal counseling/support on breastfeeding and other infant feeding practices.

Some limitations to this study may include bias in reporting (mothers’ self-report) of the primary outcome (exclusive breastfeeding). To counter this, data will be collected longitudinally at various time points, and a thorough questioning using 24-hour recall on what the baby was fed on will be done to ensure that as true as possible a record of infant feeding practices is obtained. It is possible that even with randomization of clusters; there will be differences in the baseline measures in the intervention and control groups by chance. Analysis methods to be adopted will control for baseline differences, if such differences are identified, but this will reduce the statistical power. Though we do not expect much loss to follow-up as we will work with a stable rural agrarian community, we plan to adopt intention-to-treat analysis to deal with potential loss to follow-up. Additionally, we have included an allowance for loss to follow-up in the sample size determination.

In conclusion, the importance of breastfeeding and optimal infant feeding promotion in child-survival cannot be overemphasized. Identifying feasible and effective strategies for promotion of optimal infant feeding is of utmost importance. The results from this trial will provide evidence regarding the feasibility of implementing the BFCI in Kenya and its effectiveness on breastfeeding, morbidity from diarrhea, and nutritional status among infants. This is expected to inform policy and practice regarding child survival in Kenya and other LMICs. It is expected to inform the roll-out of the BFCI in Kenya and other LIMCs where it is under consideration, which goes beyond the BFHI to promote optimal breastfeeding and other infant feeding practices at the community level.

## Trial status

The trial is currently at the formative stages. The qualitative formative study has been conducted, and the intervention has started. Recruitment of study participants is expected to end by February 2016.

## Abbreviations

ANCOVA: analysis of covariance
BFCI: Baby Friendly Community Initiative
BFHI: Baby-Friendly Hospital Initiative
CHWs: community health workers
CU: community unit
DALYs: disability-adjusted life years
FGD: focus group discussion
KEMRI: Kenya Medical Research Institute
KII: key informant interview
IDI: in-depth interview
IYCF: infant and young child feeding
IYCN: infant and young child nutrition
LAZ: length-for-age *z*-scores
LMICs: low-income and middle-income countries
MIYCN: maternal, infant and young child nutrition
MUAC: mid-upper arm circumference
NIH: National Institutes of Health
PEER: Partnership for Enhanced Engagement in Research
SCHMT: Sub-County Health Management Team
SES: socio-economic status
TBA: traditional birth attendant
UNICEF: United Nations Children’s Fund
USAID: United States Agency for International Development
WAZ: weight-for-age *z*-scores
WLZ: weight-for-length *z*-scores
WHO: World Health Organization
